# Astilbin Alleviates Radiation-Induced Pulmonary Fibrosis via *circ*PRKCE Targeting the TGF-β/Smad7 Pathway to Inhibit Epithelial–Mesenchymal Transition

**DOI:** 10.3390/biomedicines13030689

**Published:** 2025-03-11

**Authors:** Zhiling Shi, Jing Liu, Jing Qin, Xian Liang, Xue Ou, Tingting Zhang, Xueting Yan, Qianxin Hu, Weimei Huang, Kai Hu

**Affiliations:** 1 Department of Radiation Oncology, The First Affiliated Hospital of Guangxi Medical University, Nanning 530021, China; shizhiling2020@163.com (Z.S.); lemongxykdx@gmail.com (J.L.); qinjing1112@163.com (J.Q.); liangxgxmu@163.com (X.L.); ooconquer@163.com (X.O.); ztt8711@163.com (T.Z.); yxthxf7799@163.com (X.Y.); hqxxx1128@163.com (Q.H.); 2 Key Laboratory of Early Prevention and Treatment for Regional High Frequency Tumor, Guangxi Medical University, Ministry of Education, Nanning 530021, China; 3 State Key Laboratory of Targeting Oncology, Guangxi Medical University, Nanning 530021, China; 4 Department of Radiation Oncology, The Second Affiliated Hospital of Guangxi Medical University, Nanning 530007, China

**Keywords:** radiation-induced pulmonary fibrosis, astilbin, epithelial–mesenchymal transition, Smad7, radioprotection

## Abstract

**Purpose:** This study aimed to clarify the protective effect of astilbin (AST) on radiation-induced pulmonary fibrosis (RIPF) and explore its underlying molecular mechanism, focusing on non-coding RNAs. **Methods:** Mouse lung epithelial cells (MLE-12 and TC-1) and C57BL/6J mice were used to establish in vitro radiation injury models and in vivo RIPF models, respectively. Cell viability, apoptosis, the epithelial-to-mesenchymal transition (EMT), and fibrosis-related markers were assessed using cell-counting kit-8 assays, Western blotting, immunohistochemistry, and histological staining. High-throughput sequencing identified differentially expressed circRNAs. The mechanistic studies included RNA-FISH, a dual-luciferase reporter assay, an RNA immunoprecipitation (RIP) assay, and loss-of-function experiments. **Results:** AST significantly alleviated radiation-induced apoptosis and EMT in vitro, as well as RIPF in vivo. AST treatment reduced collagen deposition, fibrosis-related protein expression, and EMT marker changes. High-throughput sequencing revealed that AST upregulated *circPRKCE*, a non-coding RNA that functions through a ceRNA mechanism by binding to miR-15b-5p, thereby promoting Smad7 expression and suppressing the TGF-β/Smad7 pathway. Knockdown of *circPRKCE* abolished AST’s protective effects, confirming its pivotal role in mediating AST’s anti-fibrotic activity. **Conclusions:** This study demonstrates that Astilbin alleviates radiation-induced pulmonary fibrosis via *circPRKCE* targeting the TGF-β/Smad7 pathway to inhibit EMT, suggesting AST as a potential therapeutic agent for managing this severe complication of radiotherapy.

## 1. Introduction

Chest tumors, including lung, breast, and esophageal cancers, account for approximately 5.3 million new cases annually worldwide, representing 26.6% of all malignant tumors [1]. Radiotherapy is one of the most common treatments for thoracic tumors, playing a pivotal role in their management [2]. However, despite advances in radiotherapy, radiation-induced pulmonary fibrosis (RIPF) remains a prevalent adverse effect, impacting both treatment progression and overall life quality [3]. The mechanisms underpinning RIPF are complex and encompass multiple cell types and signaling pathways, yet effective clinical treatment strategies for RIPF are still lacking.

The epithelial-to-mesenchymal transition (EMT) is essential in embryonic development, tissue remodeling, cancer metastasis, and fibrotic diseases, and it has been established as a crucial mechanism in RIPF [4,5,6]. Studies suggest that EMT-derived mesenchymal cells constitute over 30% of effector cells in RIPF [7], and recent findings link radiation-induced EMT to the TGF-β/Smads signaling pathway. Andarawewa et al. demonstrated that treating HMEC cells with 2 Gy X-ray and exogenous TGF-β1 for 48 h induces EMT, marked by cell elongation and increased expression of N-cadherin and fibronectin [8]. In A549 cells, radiation-induced EMT is time-dependent, involving changes in cell morphology, EMT marker expression, and nuclear localization of the transcription factor snail, with TGF-β/Smad2/3 pathway activation being a key factor [9].

Research on non-coding RNAs has grown rapidly, with competitive endogenous RNA (ceRNA) recognized as a major mechanism through which non-coding RNAs function. Numerous studies report the involvement of circRNA and miRNA in the development of pulmonary fibrosis [10,11]. Yao et al. found that *circCDR1as* promotes pulmonary fibrosis in a SiO_2_-induced mouse model by sponging miR-7 and activating the TGF-β receptor [12]. Similarly, Zhang et al. reported that *circHIPK3*, upregulated in a bleomycin-induced mouse model, sponges miR-338-3p, leading to the upregulation of SOX4 and COL1A1, promoting fibrosis [13]. Additionally, miR-21 has been shown to enhance RIPF by downregulating Smad7, amplifying TGF-β signaling and increasing collagen synthesis [14].

Traditional Chinese herbal medicines, known for their dual medicinal and dietary properties, have garnered attention as radiation protectors due to their low toxicity [15]. Our research has demonstrated that extracts from S. glabra modulate the radiation-induced EMT [16]. Astilbin (AST), a pivotal flavonoid in S. glabra, exhibits diverse pharmacological activities, including anti-inflammatory [17,18], anti-oxidant [19], myocardial protection [20], regulation of metabolic disorders [21], and anti-fibrosis [22,23,24,25]. In our previous studies, both in vitro and in vivo experiments confirmed that astilbin offers protection against early radiation-induced lung injury by inhibiting p53 acetylation [26]. While these findings highlight its potential in mitigating acute damage, it remains unclear whether astilbin’s protective effects extend to the later stages of injury, particularly in the development of chronic conditions like RIPF. Therefore, this study seeks to build on our earlier work by investigating the potential of astilbin to not only prevent but also treat RIPF, addressing the critical need for effective interventions in managing this serious complication.

## 2. Materials and Methods

### 2.1. Cell Culture and Irradiation

Mouse-derived lung epithelial cell lines MLE-12 and TC-1 were acquired from the BeNa Culture Collection (Xinyang, China). The MLE-12 cells were cultured in a DMEM medium. TC-1 cells were maintained in RPMI 1640 medium. Each of the mediums was enriched with 10% fetal bovine serum (Excell Bio, Shanghai, China) and 1% antibiotics (P1400, Solarbio, Beijing, China). Cells were maintained at 37 °C in a 5% CO_2_ humidified atmosphere. MLE-12 and TC-1 cells were irradiated at a dose rate of 400 cGy/min with 4 Gy [27]. The cellular experiments were categorized into five groups: a control group (NO-IR), an irradiation group (IR), an irradiation + DMSO group (IR-DMSO), an irradiation + AST25 μg/mL group (IR-AST25), and an irradiation + AST50 μg/mL group (IR-AST50).

### 2.2. Animals and Irradiation

Female inbred *C57BL/6* mice (6–8 weeks old, 14–18 g) were provided by the Animal Experiment Center of Guangxi Medical University. All experimental protocols were approved by the Animal Care and Use Committee at Guangxi Medical University (IACUC #202410001) on 10 October 2024. All procedures were conducted in accordance with relevant institutional and national guidelines and regulations. Additionally, the study adheres to the ARRIVE guidelines for reporting animal research. According to previous studies [26], the whole chest was irradiated with 15 Gy on the eighth day of Astilbin (AST) (BIOFOUNR, Beijing, China) administration. The mice were assigned into four groups: a normal saline group (NS), an irradiation + normal saline group (IR-NS), an irradiation + AST25 mg/kg/day group (IR-AST25), and an AST25 mg/kg/day group (AST25). The tissue samples were harvested at 16 and 24 weeks after irradiation.

### 2.3. AST Treatment

Cells were treated with AST at an appropriate concentration that was dissolved in DMSO. For the animals, AST was dissolved in physiological saline and given by intragastrical administration for 7 days before irradiation. No gavage was given on the day of irradiation on the 8th day, and the gavage was continued on the 9th day for 2 weeks.

### 2.4. Western Blotting (WB)

Cells were broken down in RIPA buffer and split using a 10% SDS-PAGE gel, transferred onto PVDF membranes and incubated with the following antibodies: GAPDH (cat. no. AF7021, Affinity Biosciences, Changzhou, China), N-cadherin (cat. no. AF5239, Affinity Biosciences, Changzhou, China), E-cadherin (cat. no. AF0131, Affinity Biosciences, Changzhou, China), vimentin (cat. no. 60330-1-Ig, Proteintech Group, Wuhan, China), snail (cat. no. AF6032, Affinity Biosciences, Changzhou, China), Smad7 (cat. no. AF5147, Affinity Biosciences, Changzhou, China), COL1A2 (cat no. 14695-1-AP, Proteintech Group, Wuhan, China), and Fibronectin (cat no. 15613-1-AP, Proteintech Group, Wuhan, China). Subsequently, the samples were incubated with horseradish peroxidase-conjugated secondary antibody, including HRP-goat anti-rabbit IgG or HRP-goat anti-mouse antibody, and the results were visualized via Syngene (Cambridge, UK).

### 2.5. Real-Time RT-PCR

TRIzol reagent (Invitrogen, Carlsbad, CA, USA) was used to extract the total RNA from cells or lung tissues. The first-strand cDNA System was reversely transcribed by using MonScriptTM RTIII All-in-One Mix with dsDNase and MonScriptTM miRNA First Strand cDNA Synthesis Kit. The relative expressions of mRNA and miRNA were quantified by a MonAmpTM SYBR^®^ Green qPCR Mix Kit on CFX96 Touch Real-Time PCR Detection System (Bio-Rad, Hercules, CA, USA). Delta CT values of the target gene were normalized to *β-actin* or *U6*. And the data were evaluated by the 2^−ΔΔCt^ method.

The primers were as follows: *Smad7*: 5′-ATTTTCTCAAACCAACTGCAGGC-3′ (forward) and 5′-ACACAGTAGAGCCTCCCCAC-3′ (reverse); *mmu-miR-15b-5p*: 5′-CCTAGCAGCACATCATGGTTTACA-3′ (forward); *mmu_circ_0000831* (diverge primer)/*circPRKCE*: 5′-CTACGCTGTGAAGGTCTTGAAGAA-3′ (forward) and 5′-GGTGAACATTCATTTTGCAGACTTG-3′ (reverse); *mmu_circ_0000831* (converge primer)/*circPRKCE*: 5′-AGCCACCGTGGATTATCCG-3′ (forward) and 5′-GCCTCTTTCTACAAACACCTGC-3′ (reverse); *COL1A2*: 5′-GTCTTGCTGGCCTACATGGT-3′ (forward) and 5′-AAAGTCATAGCCACCTCCGC-3′ (reverse); *Fibronectin*: 5′-GACGTTGCAGAGCTATCCATTTC-3′ (forward) and 5′-AGTGAATGAGTTGGCGGTGATAT-3′ (reverse); *β-actin*: 5′-TGCTGTCCCTGTATGCCTCTG-3′ (forward) and 5′-TGATGTCACGCACGATTTCC-3′ (reverse); and *U6*: 5′-CTCGCTTCGGCAGCACA-3′ (forward) and 5′-AACGCTTCACGAATTTGCGT-3′ (reverse).

### 2.6. Cell Counting Kit 8 Assays (CCK-8)

The cells were plated into 96-well dishes at a density of 800 cells per well and treated with AST for various treatment times and irradiation doses. Following treatment, the cells were incubated with DMEM or RPMI 1640 medium involving 10% CCK-8 (Biosharp, Hefei, China). After incubation for 1.5 h, the absorbance at 450 nm was measured using a microplate spectrophotometer (Synergy H1M, Bio-Rad, Hercules, CA, USA). Calculate cell survival rate using the following formula: Cell survival rate (%) = (As − Ab)/(Ac − Ab) × 100, where As is experimental wells, Ac is control wells, and Ab is blank wells.

### 2.7. Histopathology

The lung tissues were encased in paraffin and sliced into sections 0.4 μm thick. Hematoxylin and eosin (HE) (G1120, Solarbio, Beijing, China) and Masson’s trichrome staining (G1346, Solarbio, Beijing, China) were performed in accordance with the manufacturer’s directions to evaluate the pathologic grade of fibrotic changes in tissues. For HE, in brief, the paraffin sections were deparaffinized with xylene and rehydrated with alcohol. The nuclei were stained with hematoxylin, followed by dyeing cytoplasm with eosin, rapidly dehydrated, air-dried, and sealed. For Masson’s trichrome staining, sections were dewaxed and hydrated, followed by immersion in a mordant solution overnight at room temperature. After rinsing, they were stained with lapis lazuli blue and hematoxylin, each for 2 min. Differentiation with acidic ethanol was brief, followed by Ponceau fuchsin staining for 10 min. Following treatment with phosphomolybdic acid, the sections were counterstained with aniline blue. Finally, they were treated with weak acid, dehydrated, and sealed. The sections were examined under an Olympus microscope.

### 2.8. Immunohistochemistry

The lung tissue sections were dewaxed and hydrated, followed by antigen retrieval using a 1× sodium citrate antigen retrieval solution (C1032, Solarbio, Beijing, China). After applying a peroxidase blocker to inactivate endogenous enzymes, the sections were blocked with 5% bovine serum albumin (SW3015, Solarbio, Beijing, China). Subsequently, the sections were incubated overnight at 4 °C with diluted primary antibodies, including COL1A2, Smad7, Fibronectin, N-cadherin, E-cadherin, vimentin, and snail. After incubation with the secondary antibody, DAB was used for color development. The sections were then counterstained with hematoxylin, dehydrated, cleared, and sealed. Ultimately, they were examined via an Olympus microscope.

### 2.9. Bioinformatics Analysis

High-throughput RNA sequencing was performed for the lung tissues of the RIPF mice model with or without AST. The differentially expressed circRNAs in the RIPF mouse model with AST treatment were screened using |logFC| > 2 and *p*-value < 0.05. The genomic annotation of *mmu-circ-0000831* (*circPRKCE*) and prediction of its downstream-targeted miRNAs were analyzed using UCSC (https://genome.ucsc.edu/, accessed on 6 March 2021) and the StarBase (http://starbase.sysu.edu.cn/, accessed on 6 June 2021) database. After identifying *miR-15b-5p* as the downstream target of the *circPRKCE*, candidate downstream mRNAs of the miRNA were predicted using databases such as StarBase (http://starbase.sysu.edu.cn/, accessed on 10 July 2021), miRDB, miRWalk (http://mirwalk.umm.uni-heidelberg.de/, accessed on 10 July 2021), and TargetScan (http://www.targetscan.org/, accessed on 10 July 2021).

### 2.10. Sanger Sequencing, RNase R Digestion, and DNA Gel Electrophoresis

In accordance with previous studies on circRNA [28], the present study utilized the following methods to verify the circular characteristics of *circPRKCE*, as detailed below. The *circPRKCE* sequence was obtained using divergent primers and sent to the Beijing Genomics Institute (Beijing, China) for Sanger sequencing analysis. Total RNA (3 μg) was incubated at 37 °C for 30 min with or without 20 U/μL RNase R (Geneseed Biotech, R0301, Guangzhou, China). The products were then reverse transcribed, and RT-qPCR was performed with the corresponding primers. The DNA gel electrophoresis was performed by loading the resulting cDNA into the wells of a 1% agarose gel submerged in 1× TAE buffer (CR00501S, Monad Biotech, Suzhou, China). The electrophoresis was conducted at a voltage of 120 V for 30 min, and the agarose gel was then developed using a gel documentation system.

### 2.11. Fluorescent In Situ Hybridization (FISH)

Based on a previous study regarding the subcellular localization of non-coding RNAs [29], the localization of *circPRKCE* in MLE-12 cells was ascertained through FISH. Briefly, MLE-12 cells were plated onto a 24-well plate. After 24 h, MLE-12 cells were subjected to fixation with 4% paraformaldehyde, and a fluorescence in situ hybridization kit was utilized to conduct the above experiment, adhering to the manufacturer’s guidelines (F16502, Genepharma, Suzhou, China). Cy3-labeled probes for *circPRKCE* and 18s were applied during hybridization. The above probes were obtained from Genepharma, (Suzhou, China). Finally, images were captured via an upright fluorescence microscope (BX53+DP80, Olympus, Tokyo, Japan).

### 2.12. RNA Immunoprecipitation (RIP) Assay

Cells were harvested from 10 cm dishes and processed utilizing an RNA immunoprecipitation (RIP) kit (Bes5101, BersinBio, Guangzhou, China). The cells were lysed in protease inhibitor, polysome lysis buffer, and ribonuclease inhibitor. Cell lysates were incubated overnight at 4 °C with Anti-Argonaute 2(AGO2) antibody (5 μg, PH1731, Abmart, Shanghai, China) or the anti-IgG antibody-conjugated beads. Before proteinase K treatment, a portion of the sample was collected into 1.5 mL tubes for WB; the rest was eluted and subjected to RT-PCR analysis.

### 2.13. Dual-Luciferase Reporter Assay

The 3′UTR binding sequences of *circPRKCE* and *smad7* were inserted downstream of the luciferase gene in the pmirGLO vector to construct wild-type *circPRKCE-WT* and *smad7-WT* plasmids. Based on the database-predicted binding sites of *circPRKCE*, *smad7*, and *miR-15b-5p*, the predicted binding sites in the *circPRKCE* and *smad7* sequences were mutated to create *circPRKCE-MUT* and *smad7-MUT* plasmids. The accuracy of these insertions was confirmed by sequencing. All plasmids were constructed by Genepharma (Suzhou, China). 293T cells were plated in 12-well plates and cultured for 24 h to achieve 70–80% confluence. Subsequently, 2 μg of the constructed luciferase reporter plasmids, along with *miR-15b-5p mimic* or *mimic-NC*, were co-transfected into the cells for 48 h using Lipofectamine 3000 (L3000015, Invitrogen, Carlsbad, CA, USA). Luciferase activity was measured using a Dual-Luciferase Reporter Assay kit (E1910, Promega, Madison, WI, USA). The activity was quantified by comparing the Firefly luciferase activity to the Renilla luciferase activity ratio.

### 2.14. Construction of Stable-Infected Cell Lines

A mouse lentivirus containing *sh-circPRKCE* was designed and obtained from HanBio (Shanghai, China). After determining the appropriate multiplicity of infection (MOI) and optimal puromycin concentration, MLE-12 and TC-1 cells were transduced with the concentrated lentiviral particles. Cells were chosen in a medium supplemented with 6 μg/mL puromycin 48 h post-transfection.

### 2.15. Flow Cytometry

The Annexin V-APC/7-AAD Apoptosis Kit (MultiSciences Biotech Co., Ltd., Hangzhou, China) was used for the apoptosis analysis of mouse lung epithelial cells among the different treatment groups. After digestion, resuspend the cells with 500 μL 1 × binding buffer. Then, every group added 5 μL Annexin V-APC and 10 μL 7-AAD, and the mixtures were incubated at room temperature for 5 min in the dark. The apoptosis rate was assessed by utilizing flow cytometry (Accuri C6 Plus, BD, Franklin Lakes, NJ, USA).

### 2.16. Statistical Analysis Doses and Times

All continuous variables were analyzed using SPSS 26.0 and are presented as the mean ± standard deviation (*mean* ± *SD*). For a quantitative data analysis of multiple groups, variance analysis was performed after confirming the homogeneity of variance between the groups. The Student’s *t*-test was used to compare the same indicator between two groups, following a normality test to ensure normal distribution. Data visualization and additional analysis were conducted using GraphPad Prism (version 8.3.0, GraphPad Software, San Diego, CA, USA) and ImageJ (version 1.51j8, National Institutes of Health, Bethesda, MD, USA). A *p*-value < 0.05 was considered statistically significant.

## 3. Results

### 3.1. AST Reduces Radiation-Induced Inhibition of Proliferation in Mouse-Derived Lung Epithelial Cells In Vitro

**Figure 1:**
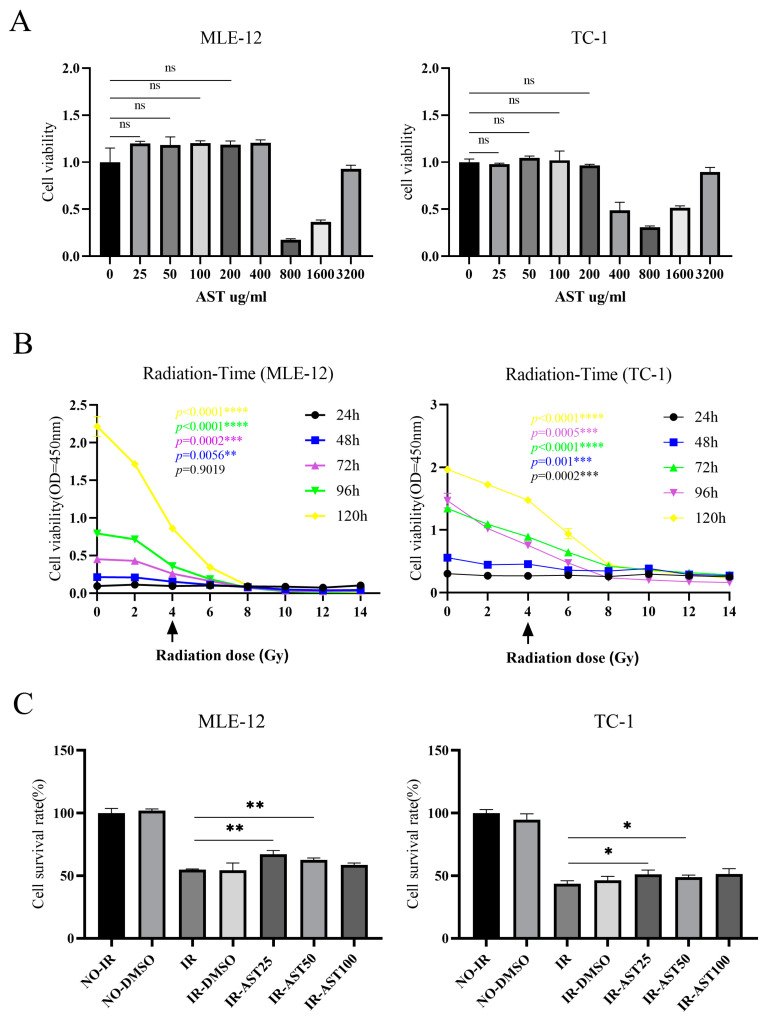
AST reduces radiation-induced apoptosis in mouse-derived lung epithelial cells in vitro. (**A**) Cell viability of MLE-12 and TC-1 cells was assessed by CCK8 assay under varying concentrations of AST. (**B**) Cell viability of MLE-12 and TC-1 cells was measured following different irradiation doses (4 Gy–14 Gy) at multiple time points (24 h–120 h). (**C**) Cell survival rate was evaluated in MLE-12 and TC-1 cells treated with different concentrations of AST under a 4 Gy irradiation dose for 48 h. (IR-AST25, IR-AST50, and IR-AST100 represent AST at 25, 50, and 100 µg/mL, respectively). All data are presented as *mean* ± *SD* (*n* = 3). ns: not significant; * *p* < 0.05; ** *p* < 0.01; *** *p* < 0.001; **** *p* < 0.0001.

In this study, we explored the effects of astilbin (AST) on radiation-induced injury in mouse lung epithelial cells using the MLE-12 and TC-1 cell lines. We found that high concentrations of AST significantly reduced cell viability in both MLE-12 and TC-1 cells under non-irradiated conditions. However, when the AST concentration was below 200 µg/mL, cell viability either increased slightly or no significant damage was observed (Figure 1A). These findings suggest that AST concentrations below 200 µg/mL are safe for use with mouse lung epithelial cells in vitro.

Next, we assessed cell viability in MLE-12 and TC-1 cells at multiple time points following exposure to varying radiation doses. In the 4 Gy irradiation group, both cell lines exhibited significant cell death, particularly between 48 and 120 h post-irradiation (Figure 1B). Based on these conditions, we further investigated whether AST could protect lung epithelial cells from radiation-induced inhibition of proliferation. Our results showed that AST concentrations of 25 µg/mL and 50 µg/mL significantly reduced inhibition of proliferation in MLE-12 and TC-1 cells after 48 h of 4 Gy irradiation (Figure 1C). Together, these findings indicate that AST provides protective effects against radiation-induced damage in lung epithelial cells in vitro.

### 3.2. AST Alleviates Radiation-Induced Pulmonary Fibrosis In Vivo

**Figure 2:**
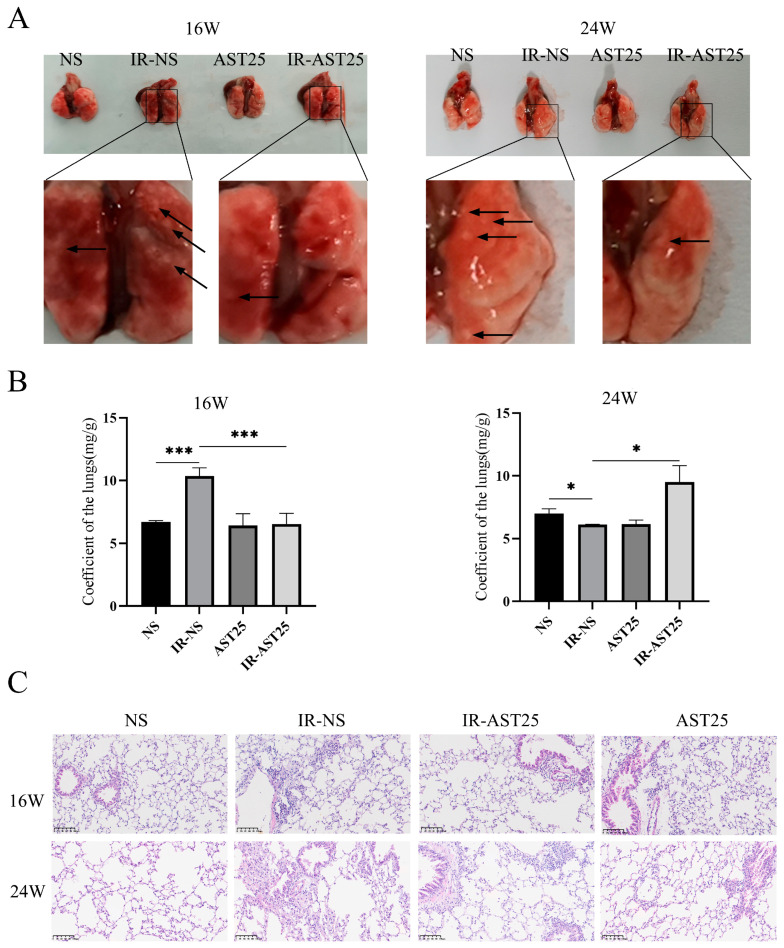
AST alleviates radiation-induced pulmonary fibrosis in vivo. (**A**) Representative gross images of mouse lungs at 16- and 24-weeks post-treatment. (The black arrow indicates the lesion site within the gross lung tissue). (**B**) Lung weight-to-body weight ratio at 16 and 24 weeks. (**C**) H&E staining of lung tissue sections from each group at 16 and 24 weeks. Scar bar: 100 μm. Treatment groups: NS (non-irradiated, normal saline only); IR-NS (irradiated with 15 Gy, normal saline); IR-AST25 (irradiated with 15 Gy, 25 mg/kg AST); AST25 (non-irradiated, 25 mg/kg AST). All data are shown as *mean* ± *SD* (*n* = 3). * *p* < 0.05; *** *p* < 0.001.

Our previous study demonstrated that AST alleviates radiation-induced lung injury (RILI) in *C57BL/6J* mice [26], and since RILI is an early pathological stage of radiation-induced pulmonary fibrosis (RIPF) [30], we next investigated whether AST also exerts protective effects on RIPF in vivo. To model RIPF, mice were irradiated with 15 Gy and treated with or without AST for 16 and 24 weeks (Figure 2A). At 16 weeks, the lung weight-to-body weight ratio was significantly reduced in the AST treatment group compared to the IR-NS group. However, this effect was reversed at 24 weeks (Figure 2B). These findings suggest that AST may help reduce early inflammation following pulmonary irradiation and alleviate late-stage pulmonary fibrosis.

**Figure 3:**
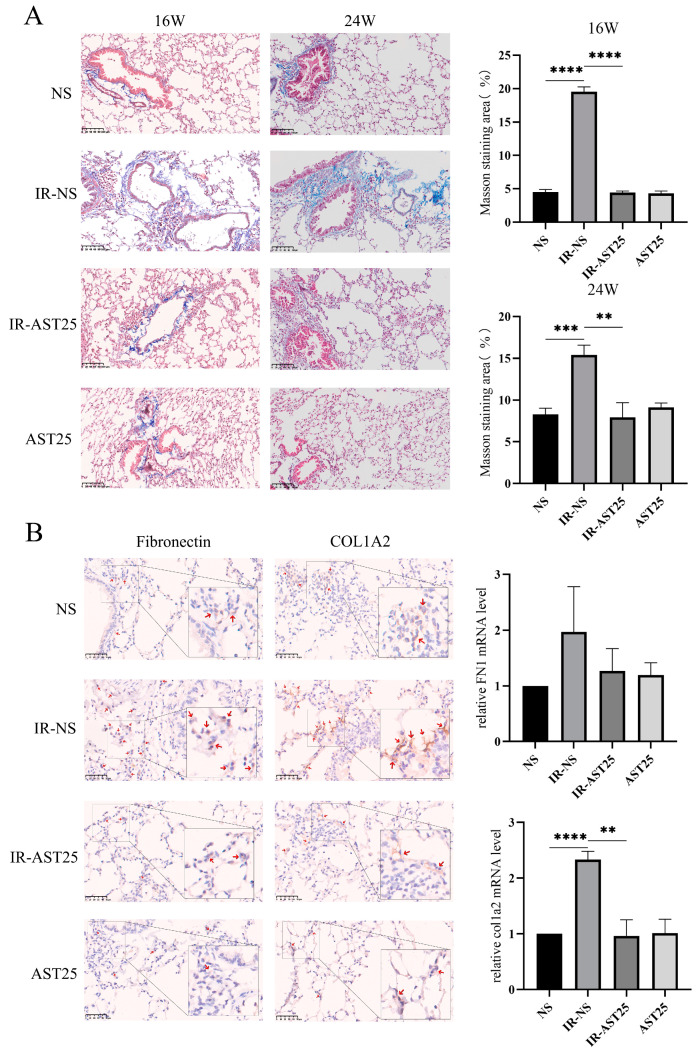
AST alleviates radiation-induced pulmonary fibrosis in vivo. (**A**) Masson’s trichrome staining (**left**) and quantification of fibrotic area (**right**) in mouse lung tissue sections at 16 and 24 weeks. Scar bar: 100 μm. (**B**) Immunohistochemical staining (**left**) and qRT-PCR (**right**) analysis of Fibronectin and COL1A2 expression at 24 weeks. (The red arrows indicate the prominent positive staining areas). Scar bar: 50 μm. Treatment groups: NS (non-irradiated); IR-NS (irradiated with 15 Gy); IR-AST25 (irradiated with 15 Gy, 25 mg/kg AST); AST25 (25 mg/kg AST only). All data are shown as *mean* ± *SD* (*n* = 3). ** *p* < 0.01; *** *p* < 0.001; **** *p* < 0.0001.

Histological analysis revealed increased infiltration of inflammatory cells and more pronounced thickening of alveolar and bronchiolar walls in the lung tissue of irradiated mice (IR group) compared to non-irradiated controls (NS group). AST treatment partially mitigated these effects (Figure 2C). Additionally, Masson’s Trichrome staining showed a significant increase in the area of blue-dyed collagen fibers in the IR group at both 16 and 24 weeks, which was reduced with AST treatment (Figure 3A). Similarly, increased expression of fibrosis-related proteins, such as Fibronectin and COL1A2, in the irradiated lungs was attenuated by AST (Figure 3B). In conclusion, these results suggest that AST alleviates RIPF in vivo.

### 3.3. AST Inhibits Radiation-Mediated Epithelial-to-Mesenchymal Transition Progression

**Figure 4:**
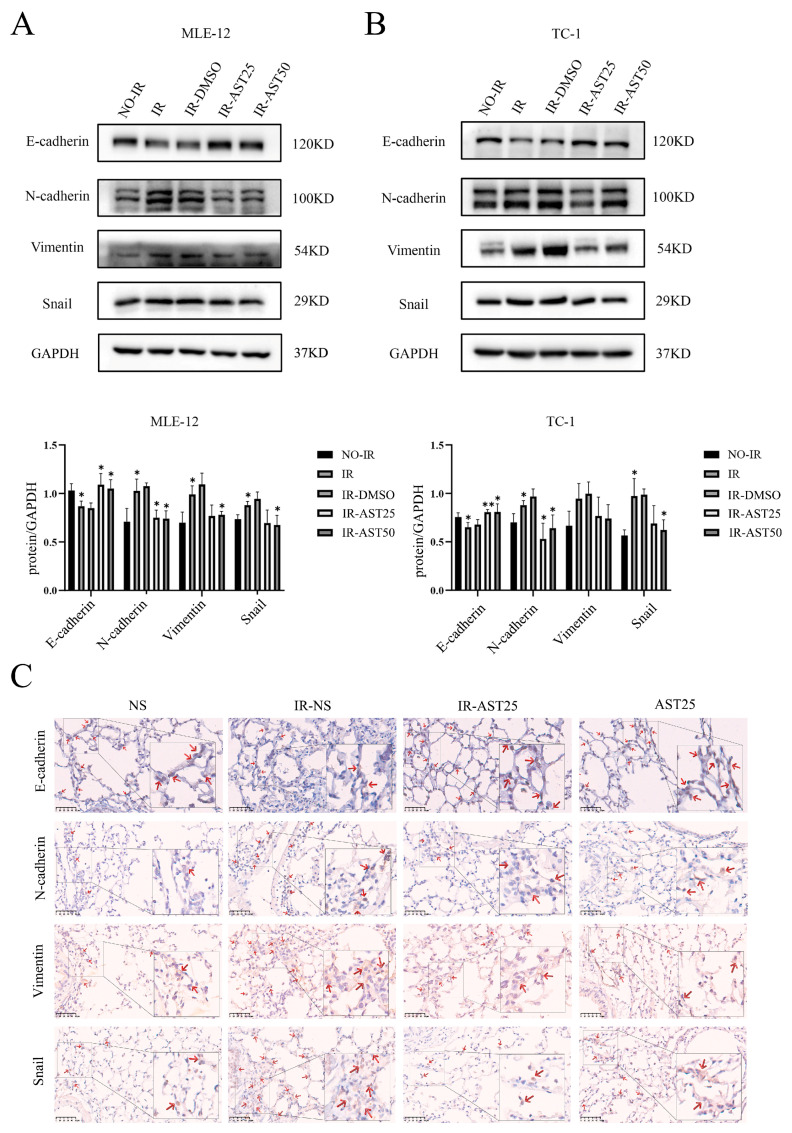
AST inhibits radiation-induced epithelial-to-mesenchymal transition progression. (**A**,**B**) Expression levels of epithelial (E-cadherin) and mesenchymal markers (N-cadherin, vimentin, and snail) were evaluated by Western blotting in MLE-12 (**left**) and TC-1 (**right**) cells. MLE-12 and TC-1 cells were irradiated with 4 Gy for 48 h with or without AST (25 µg/mL or 50 µg/mL). (**C**) Immunohistochemical staining of E-cadherin, N-cadherin, vimentin, and snail proteins was performed on mouse lung tissues from the indicated treatment groups. (The red arrows indicate the prominent positive staining areas). Scar bar: 50 μm. The treatment groups were NS (saline only); IR-NS (15 Gy irradiation + saline); IR-AST25 (15 Gy irradiation + 25 mg/kg AST); AST25 (25 mg/kg AST only). All data are shown as *mean* ± *SD* (*n* = 3). * *p* < 0.05; ** *p* < 0.01.

EMT plays a key role in the pathological fibrosis of various tissues and organs [6]. Research has demonstrated that overexpression of EMT-related genes or radiation-induced EMT accelerated the formation of RIPF [31,32]. We thus investigated whether AST affects the EMT-like process in lung tissues caused by radiation. We first detected the expression of EMT-related protein in lung epithelial cells. Western blot results confirmed that irradiation (IR) decreased the expression of the epithelial marker E-cadherin while increasing that of the mesenchymal markers including N-cadherin, vimentin, and snail. However, combined AST treatment restored the IR-induced changes in the expression of these EMT-related markers to a certain extent (Figure 4A,B). In addition, immunochemistry analysis of E-cadherin, N-cadherin, vimentin, and snail expression in irradiation-exposed mouse lung tissues also showed a similar pattern to that observed in the Western blot analysis of cellular level (Figure 4C). These observations point to AST alleviating the progression of EMT in irradiated mouse lung epithelial cells and lung tissue.

### 3.4. AST Inhibits Radiation-Induced Pulmonary Fibrosis by Enhancing circPRKCE Expression

**Figure 5:**
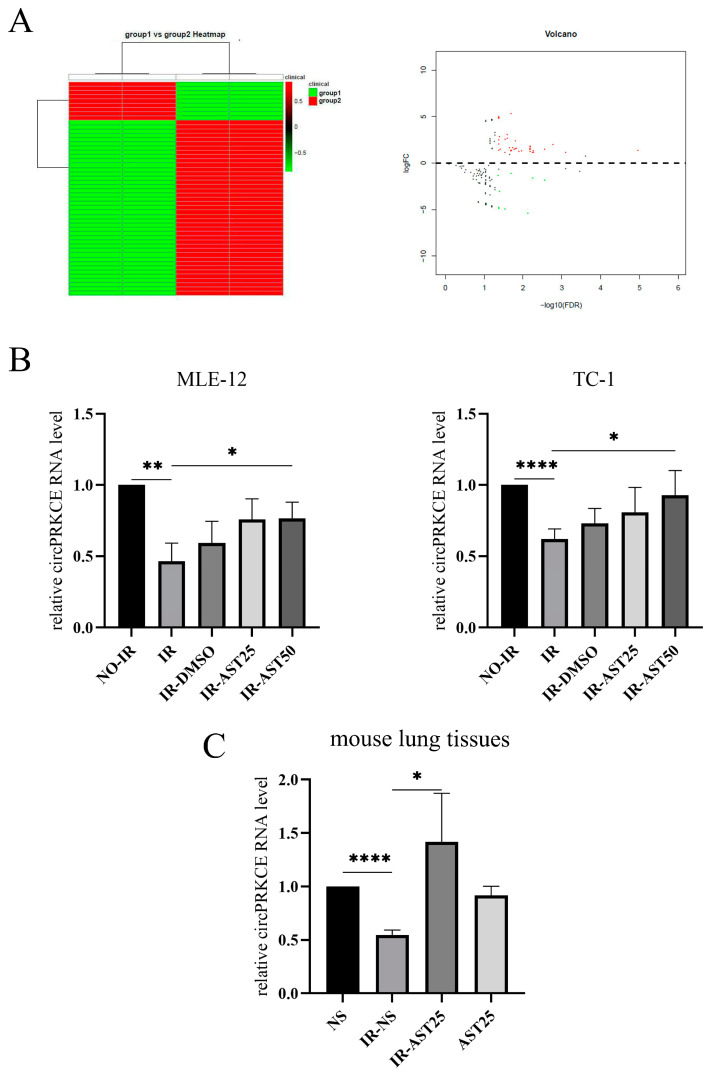
AST inhibits radiation-induced pulmonary fibrosis by enhancing *circPRKCE* expression. (**A**) High-throughput sequencing was used to analyze the differentially expressed circRNAs in irradiated lung tissue from mice treated with or without AST (group 1: no AST, group 2: with AST) (Red dots: significant upregulation; green dots: significant downregulation). (**B**) Expression of *circPRKCE* in mouse lung epithelial cells (MLE-12 and TC-1) from the indicated treatment groups. (**C**) qRT-PCR analysis of *circPRKCE* expression in mouse lung tissues from different groups (*n* = 3 per group). All data are shown as *mean* ± *SD* (*n* = 3). * *p* < 0.05; ** *p* < 0.01; **** *p* < 0.0001.

Recent research has demonstrated that circRNAs are crucial in the progression of organ fibrosis [10]. In this study, we used high-throughput sequencing to identify differentially expressed circRNAs in the irradiated lung tissue of mice treated with or without AST (Figure 5A). Our results revealed a significant increase in the expression of *mmu-circ-0000831 (circPRKCE)* in the lungs of AST-treated mice. *CircPRKCE*, a 614-nt long RNA, is located at the *PRKCE* gene locus on chromosome 17. Further in vitro experiments confirmed that *circPRKCE* expression was decreased in irradiated lung epithelial cells but increased upon AST treatment (Figure 5B). These in vitro findings were corroborated by in vivo experiments (Figure 5C), suggesting that AST may exert a protective effect on the lungs by regulating *circPRKCE*.

**Figure 6:**
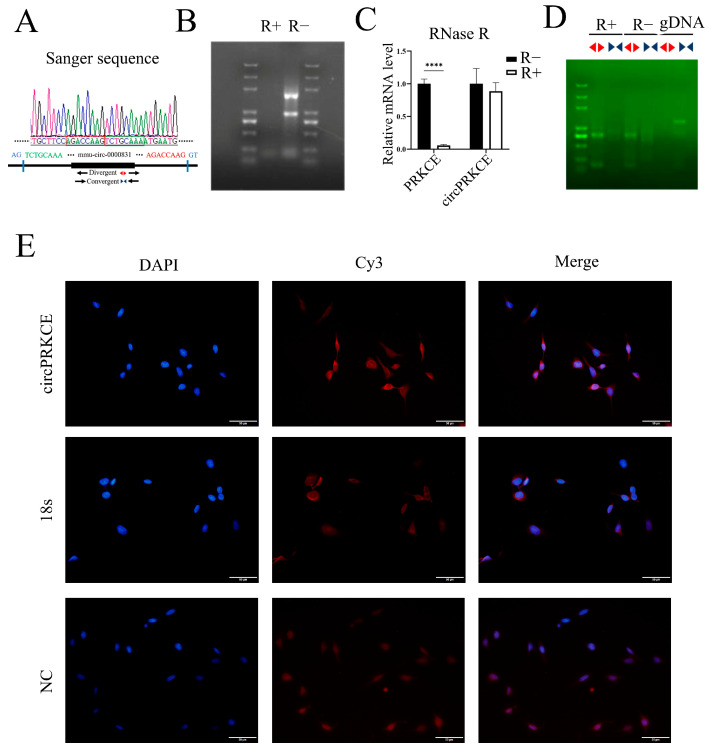
Identification of the circular nature and cellular localization of *circPRKCE*. The following experiments were performed using MLE-12 cells. (**A**) Divergent primers were used to detect total (circular) RNAs, and Sanger sequencing confirmed head-to-tail splicing. (**B**) RNase R digestion efficiency was verified by gel electrophoresis. (**C**) qRT-PCR analysis following RNase R digestion. (**D**) DNA gel electrophoresis of qRT-PCR products (including gDNA group). (**E**) RNA fluorescence in situ hybridization of *circPRKCE* (red) in MLE-12 cells. DAPI staining (blue) shows the nuclei. R+: RNase R+; R−: RNase R−; *PRKCE: lncPRKCE*; *gDNA*: genomic DNA; 18s (red): cytoplasmic expression, used as a positive control; NC: negative control. Scar bar: 50 μm. Magnification: 40×. All data are shown as *mean* ± *SD* (*n* = 3). **** *p* < 0.0001.

To verify this hypothesis, we first confirmed the circular structure of *circPRKCE* and its localization in mouse lung epithelial cells. The head-to-tail splicing sequence was identified by comparing the sequencing data with the *circPRKCE* sequence in circBASE using DNAMAN (Figure 6A). We then assessed *circPRKCE’s* resistance to RNase R, an exonuclease that digests linear RNA (Figure 6B). Following RNase R treatment, qRT-PCR with divergent and convergent primers showed that, while the linear *lncPRKCE* levels significantly decreased, the *circPRKCE* levels remained unchanged (Figure 6C). DNA gel electrophoresis further confirmed these findings, with detectable bands for *circPRKCE* in the R+ group using divergent primers, but none with convergent primers (Figure 6D), indicating *circPRKCE’s* circularity and RNase R resistance. RNA-FISH analysis revealed that *circPRKCE* is predominantly localized in the cytoplasm (Figure 6E), suggesting it likely functions through a ceRNA mechanism in lung epithelial cells [33].

**Figure 7:**
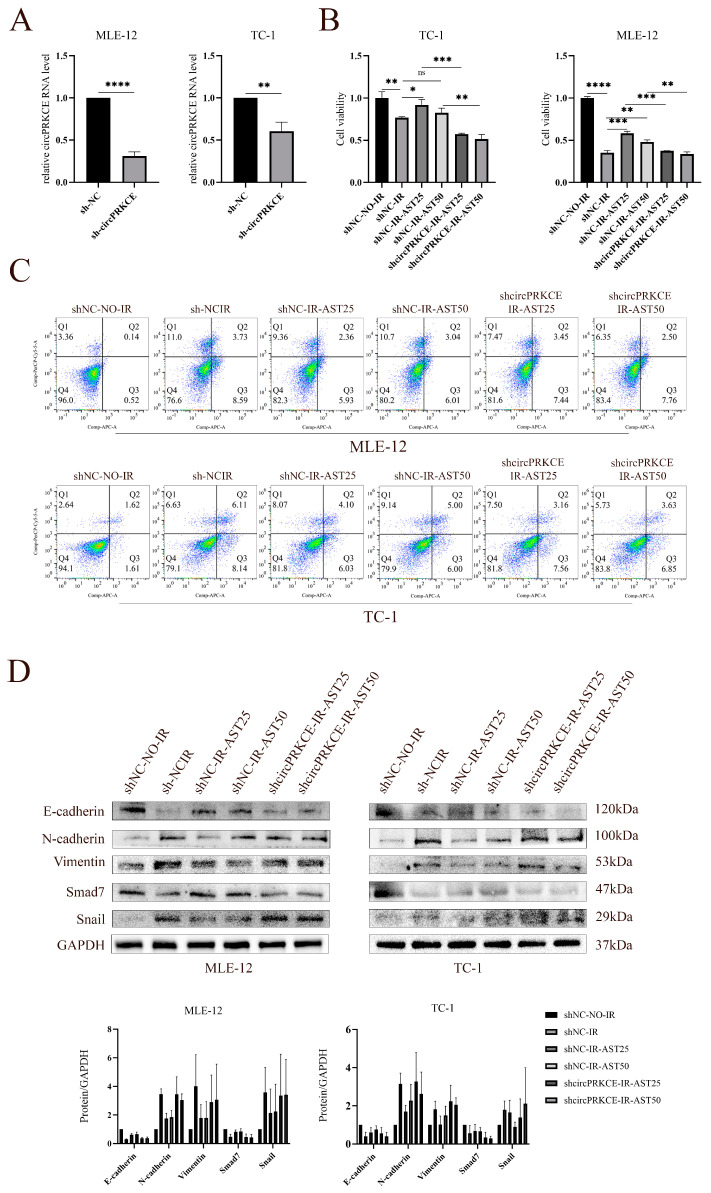
Downregulation of *circPRKCE* counteracts the radioprotective effect of AST on mouse lung epithelial cells. (**A**) Silencing efficiency of *circPRKCE* was confirmed by qRT-PCR. (**B**,**C**) CCK8 assay and flow cytometry were used to assess cell proliferation and confirm the impact of *circPRKCE* downregulation on AST-mediated radioprotection. (**D**) The effect of *circPRKCE* knockdown on AST’s ability to reverse radiation-induced EMT progression was evaluated by Western blotting. *Mmu-circ-0000831: circPRKCE*. All data are shown as *mean* ± *SD* (*n* = 3). ns: not significant; * *p* < 0.05; ** *p* < 0.01; *** *p* < 0.001; **** *p* < 0.0001.

Next, we explored the regulatory role of AST on *circPRKCE* in RIPF. In a loss-of-function experiment, we silenced *circPRKCE* using shRNA, as shown in Figure 7A. CCK8 and flow cytometry assays revealed that *circPRKCE* downregulation negated AST’s radioprotective effect on lung epithelial cells (Figure 7B,C). Additionally, immunoblotting showed that *circPRKCE* knockdown counteracted AST’s suppression of radiation-induced EMT, as indicated by increased mesenchymal markers and decreased epithelial markers (Figure 7D). These results suggest that AST mitigates RIPF by upregulating *circPRKCE* in lung epithelial cells.

### 3.5. AST Inhibited Radiation Induced Epithelial-to-Mesenchymal Transition by Regulating circPEKCE/miR-15b-5p/smad7 Axis

**Figure 8:**
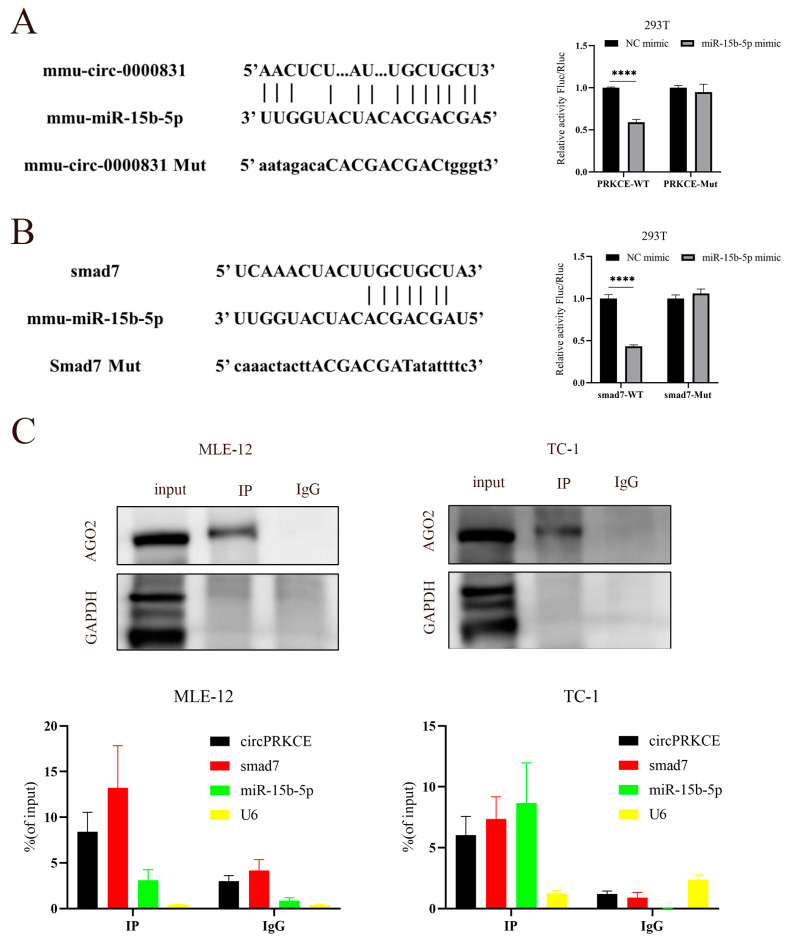
AST inhibits radiation-induced epithelial-to-mesenchymal transition by regulating the *circPRKCE/miR-15b-5p/Smad7* axis. (**A**) Bioinformatic prediction of *miR-15b-5p* binding sites in the 3′-UTR of *circPRKCE* (**left**). Dual-luciferase assay analysis of luciferase activity in 293T cells (**right**). (**B**) Bioinformatic prediction of *miR-15b-5p* binding sites in the 3′-UTR of Smad7 (**left**). Luciferase activity in 293T cells was measured by dual-luciferase assays (**right**). (**C**) AGO2 antibody pull-down effect in RIP assays in MLE-12 and TC-1 cells via Western blotting (**top**). RIP-derived RNA was detected by qRT-PCR to verify the interaction between *circPRKCE*, *miR-15b-5p*, and *Smad7* in MLE-12 and TC-1 cells (**bottom**). *Mmu-circ-0000831*: *circPRKCE*; WT: wild type; Mut: mutant; IP: AGO2 antibody immunoprecipitation; IgG: IgG antibody control. All data are shown as *mean* ± *SD* (*n* = 3). **** *p* < 0.0001.

Our previous results showed that *circPRKCE* is localized in the cytoplasm of epithelial cells. Previous studies have demonstrated that cytoplasmic non-coding RNAs (ncRNAs) regulate gene expression through the competing endogenous RNA (ceRNA) mechanism [33], suggesting that *circPRKCE* may participate in regulating the RIPF process via this pathway. In this study, bioinformatics analysis identified *mmu-miR-15b-5p* as a potential downstream target of *circPRKCE*. As predicted, dual-luciferase assays in 293T cells confirmed the binding interaction between *circPRKCE* and *mmu-miR-15b-5p* (Figure 8A). Interestingly, both bioinformatics predictions and dual-luciferase experiments also revealed a physical binding relationship between *mmu-miR-15b-5p* and *Smad7* (Figure 8B). Furthermore, a RIP experiment using AGO2, a key protein of the RNA-induced silencing complex (RISC), as bait, demonstrated the interaction among *circPRKCE*, *mmu-miR-15b-5p*, and *Smad7* (Figure 8C).

**Figure 9:**
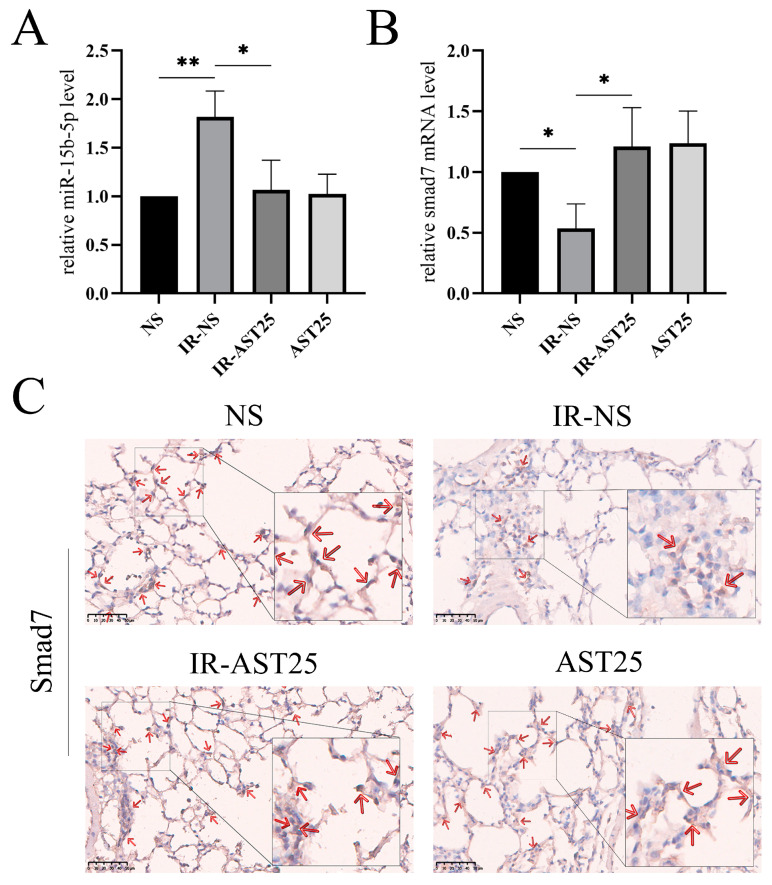
AST mitigates radiation-induced epithelial-to-mesenchymal transition via *circPRKCE* targeting the TGF-β/Smad7 pathway. (**A**) qRT-PCR analysis showing the expression of *miR-15b-5p* in mouse lung tissues across different treatment groups. (**B**,**C**) Expression levels of Smad7 in mouse lung tissues across groups, assessed via qRT-PCR and immunohistochemical staining. (The red arrows indicate the prominent positive staining areas). Scar bar: 50 μm. Data are presented as *mean* ± *SD* (*n* = 3). Statistical significance: ns: not significant; * *p* < 0.05; ** *p* < 0.01.

**Figure 10:**
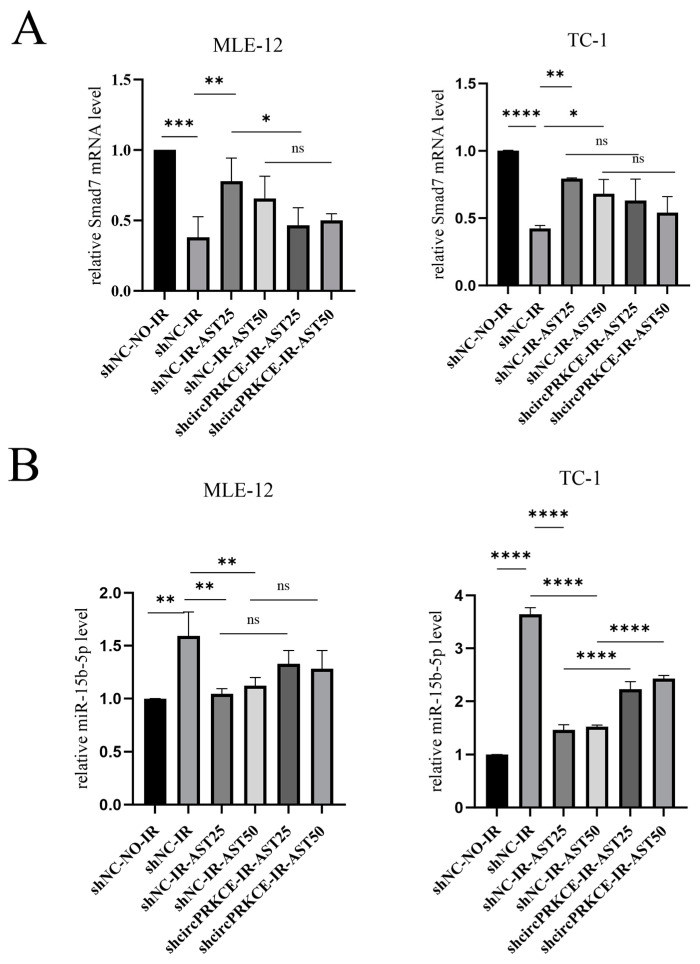
AST mitigates radiation-induced epithelial-to-mesenchymal transition via *circPRKCE* targeting the TGF-β/Smad7 pathway. (**A**,**B**) Downregulation of *circPRKCE* reversed the regulatory effects of AST on *mmu-miR-15b-5p* and *Smad7* in irradiated epithelial cells. *Mmu-circ-0000831*: *circPRKCE*. Data are presented as *mean* ± *SD* (*n* = 3). Statistical significance: ns: not significant; * *p* < 0.05; ** *p* < 0.01; *** *p* < 0.001; **** *p* < 0.0001.

Smad7 is known as a negative regulator of EMT [34]. Drawing on these findings, we postulate that the *circPRKCE/miR-15b-5p/Smad7* axis may be the mechanism through which AST exerts its protective effects against RIPF. To further investigate this, we conducted qRT-qPCR and immunohistochemistry experiments. The results indicated that the expression level of *mmu-miR-15b-5p* was significantly increased in irradiated mouse lung tissues but decreased following AST treatment (Figure 9A). Conversely, the expression of Smad7 was significantly reduced in irradiated lung tissues, while it increased upon AST treatment (Figure 9B,C). Additionally, we found that the downregulation of *circPRKCE* reversed the regulatory effects of AST on *mmu-miR-15b-5p* and *Smad7* in irradiated epithelial cells (Figure 10A,B). In summary, these results suggest that AST inhibits radiation-induced EMT by regulating the *circPRKCE/miR-15b-5p/Smad7* axis.

## 4. Discussion

Radiation-induced pulmonary fibrosis (RIPF) is a chronic, progressive, and often irreversible complication following radiotherapy for chest tumors, underscoring the importance of preventive strategies. Existing preclinical studies have shown that the multi-kinase inhibitor nintedanib, which targets growth factors like vascular endothelial growth factor, has shown promise in preventing radiation pneumonitis (RP) and reducing the incidence of pulmonary fibrosis [35]. Another emerging therapeutic, pirfenidone, has been observed to alleviate RIPF by targeting growth factors and downregulating procollagen levels [35]. Although both nintedanib and pirfenidone are currently approved for treating idiopathic pulmonary fibrosis (IPF), their efficacy in RIPF has not been established, and effective clinical treatments for RIPF remain scarce.

Astilbin (AST), a key component in traditional Chinese medicine, has been studied for its anti-fibrotic and anti-inflammatory properties [17,18,22,23,24,25]. Zhang et al. reported that the Erhuang Formula, which contains AST, reduced inflammation and fibrosis in a rat model of adenine-induced renal failure [36]. Chen et al. found that AST alleviated renal epithelial-to-mesenchymal transition (EMT) induced by indophenol sulfate via the AhR/Snai1 pathway [24]. Sun et al. showed AST reduced carbon tetrachloride-induced liver fibrosis in rats through Nrf2 activation [25], and Zhang et al. demonstrated that AST inhibited TGF-β and bleomycin-induced pulmonary fibrosis by blocking the Hedgehog pathway [23]. In our study, we found that AST effectively inhibited radiation-induced EMT in mouse lung epithelial cells, both in vivo and in vitro, reducing radiation-induced cell damage and lung fibrosis. Treatment with AST significantly decreased radiation-induced apoptosis in these cells. Additionally, AST partly reversed the irradiation-induced thickening of the alveolar and bronchiolar walls in mouse lung tissue. The AST-treated mouse showed a notably smaller Masson-stained blue area, indicating reduced fibrosis compared to the IR group. In addition, AST reduced the expression of the fibrosis-related proteins Fibronectin and COL1A2, suggesting AST’s potential in mitigating RIPF.

EMT is pivotal in tissue and organ fibrosis, and researches link RIPF development to EMT processes, which can be mitigated [37,38] or exacerbated [39] by regulating EMT. PARK et al. showed that radiation triggers EMT in mouse lung epithelial cells through M2 macrophages secreting TGF-β, reducing E-cadherin and increasing mesenchymal markers like N-cadherin and vimentin [40]. Inhibiting TGF-β/Smads signaling can alleviate RIPF, as shown by Ying et al., who found that pirfenidone reduced RIPF by limiting M2 macrophage infiltration and blocking TGF-β/Smad3 activation [41]. Similarly, Park et al. found that the herbal extract PM014 mitigated RIPF by regulating NF-κB and TGFβ1/NOX4 pathways [42]. Downstream regulators of TGF-β/Smads, such as Smad2/3 (key mediators of fibrosis) and Smad6/7 (negative regulators), play essential roles. Ren et al. reported that tacrolimus alleviates paraquat-induced pulmonary fibrosis by modulating TGF-β1, Smad3, cTGF, and Smad7 levels [43]. In our current study, radiation reduced the epithelial markers and increased the mesenchymal markers in vitro, with AST treatment reversing these changes. Immunohistochemical analysis in irradiated mouse lung tissues treated with AST showed similar patterns, suggesting that AST alleviates RIPF by inhibiting EMT through the TGF-β/Smads pathway.

AST can alleviate RIPF by influencing EMT, though the specific mechanisms, particularly within the TGF-β/Smads pathway, remain unclear. With advancements in high-throughput sequencing, circRNAs linked to fibrosis in the heart [44], liver [45], kidney [46], and lungs [47] have gained attention for their potential therapeutic role in fibrosis and aging. Song et al. identified circRNAs, specifically *circRNA-662* and *-949*, as potential AST targets in bleomycin-induced pulmonary fibrosis; these act as *miR-29b* sponges to regulate key factors like STAT3 and Gli2 in fibrosis [22]. However, circRNAs in RIPF remain unexamined. In this study, high-throughput sequencing revealed a significant increase in *mmu-circ-0000831* (*circPRKCE*) expression in AST-treated mouse lung tissues. Further experiments confirmed that AST countered the irradiation-induced decrease in *circPRKCE* expression. Sanger sequencing and RNase R digestion verified *circPRKCE* as a circular RNA formed by back-splicing. Loss-of-function studies in lung epithelial cells showed that downregulating *circPRKCE* negated AST’s protective effect on radiation-induced EMT in lung epithelial cells. These findings indicate that AST may alleviate RIPF by promoting *circPRKCE* expression in epithelial cells, offering insight into a novel regulatory mechanism of AST in RIPF.

In our study, further RNA-FISH experiments revealed that *circPRKCE* is predominantly located in the cytoplasm, suggesting it may function mainly through the ceRNA mechanism [33]. Prior research has documented that the miR-15 family regulates fibrosis across various organs, primarily by influencing the TGF-β signaling pathway [48,49,50]. RIPF is similarly modulated by multiple miRNAs [51]. Through bioinformatics analysis, dual-luciferase assay, and RIP experiments, we identified physical binding interactions between *circPRKCE* and *mmu-miR-15b-5p*, as well as between *mmu-miR-15b-5p* and *smad7*. Additionally, the qPCR and immunohistochemistry results indicated that AST inhibits the radiation-induced increase in *mmu-miR-15b-5p* expression in lung tissue while promoting the opposite trend in *smad7* expression. Crucially, we found that the downregulation of *circPRKCE* negated AST’s regulatory effects on *mmu-miR-15b-5p* and smad7 in irradiated epithelial cells. Collectively, our findings suggest that AST alleviates RIPF via *circPRKCE* targeting the TGF-β/Smad7 pathway and inhibiting radiation-induced EMT.

Our study, rooted in traditional Chinese medicine, takes an innovative approach to targeting RIPF, for which effective preventive and therapeutic options are currently lacking. We confirmed that AST has a protective effect against RIPF and uncovered its molecular mechanism of action, focusing on a unique class of non-coding RNA, circRNA. This finding opens new avenues for developing preventive and therapeutic drugs for RIPF and advancing our understanding of its underlying mechanisms. However, this study has certain limitations. First, as noted in the literature, AST has poor bioavailability and requires low-temperature storage, which may hinder its clinical application [52]. Further technical solutions, such as zein nanoparticle formulation, may help mitigate these issues. Second, while we demonstrated AST’s protective effects on RIPF, its impact on the efficacy of radiotherapy in tumor tissues within the irradiated area remains undetermined. These questions require further investigation.

## 5. Conclusions

In summary, our research indicates that astilbin (AST), a natural flavonoid derived from traditional Chinese medicine, effectively alleviates radiation-induced pulmonary fibrosis (RIPF) via *circPRKCE* targeting the TGF-β/Smad7 pathway and inhibiting radiation-induced EMT. This investigation suggests that AST is a promising agent for preventing and managing this severe radiotherapy complication.

## Data Availability

The original contributions presented in the study are included in the article, further inquiries can be directed to the corresponding author.

## Abbreviations

RIPF, radiation-induced pulmonary fibrosis; AST, Astilbin; EMT, epithelial-to-mesenchymal transition; ceRNA, competitive endogenous RNA; WB, Western blotting; CCK-8, cell counting kit 8 assays; FISH, fluorescent in situ hybridization; RIP, RNA immunoprecipitation; AGO2, anti-Argonaute 2; MOI, multiplicity of infection; RILI, radiation-induced lung injury; ncRNAs, non-coding RNA.
